# The role of stem cells in precision medicine: next-generation cancer treatment

**DOI:** 10.1186/s43046-025-00328-5

**Published:** 2025-11-02

**Authors:** Hafsa Iqbal, Bibi Khadija

**Affiliations:** 1 Quaid-i-Azam University, Islamabad, Pakistan; 2 National Skills University, Islamabad, Pakistan

**Keywords:** Precision medicine, Mesenchymal cells, Next generation sequencing (NGS)

## Abstract

**Background:**

Precision medicine has transformed oncology by tailoring treatments to the molecular and genetic characteristics of individual tumors. Stem cell-based strategies hold unique potential to complement these approaches by enabling regenerative support, targeted delivery of therapeutics, and novel models for drug screening.

**Methods:**

This review synthesizes current evidence on the integration of stem cell biology into precision cancer therapy, highlighting advances in tumor profiling, next-generation sequencing (NGS), and genome editing that enable personalized interventions.

**Results:**

Emerging applications include engineered stem cells for selective delivery of oncolytic agents, immune modulation through stem cell–derived platforms, and the use of induced pluripotent stem cells (iPSCs) for modeling tumor heterogeneity. Advances in NGS are accelerating tumor-specific profiling, facilitating gene editing of stem cells, and refining patient selection for therapy.

**Challenges:**

Despite progress, translational barriers remain, including risks of tumorigenicity, ethical concerns, high costs, immune rejection, and limited large-scale clinical validation.

**Conclusion:**

Stem cell–based precision oncology is a rapidly evolving field with significant promise. Future directions include integrating NGS-driven tumor profiling with engineered stem cells, optimizing safety through gene-editing technologies, and advancing clinical trials to establish efficacy. These efforts could reshape the landscape of individualized cancer care.

## Introduction

Cancer is still among the top contributors to mortality worldwide, with the complexity of tumor biology, heterogeneity, and resistance to conventional treatments posing significant therapeutic challenges [1–4]. A recent update from GLOBOCAN revealed that in 2022, the new cancer cases reached about 20 million and deaths from cancer reached 9.7 million [5]. Over the past decade, advancements in precision medicine have facilitated highly personalized treatments targeting the particular genetic and molecular profiles of individual tumors [6–10]. Despite advances in chemotherapy, radiotherapy, and immunotherapy, treatment responses often vary due to the genetic and molecular heterogeneity of tumors. This issue has propelled the emergence of precision medicine, a methodology that personalizes treatments, using the information of specific patients in terms of genomic, transcriptomic, and proteomic measurements [11]. The leading edge of new-generation cancer treatments are the stem cells owing to their extraordinary ability to self-renew and differentiate. The discovery of patient-specific cancer models with the targeting of cancer stem cells (CSCs) and induced pluripotent stem cells (iPSCs), and the administration of precision drugs have radically transformed various fields of medicine, including oncology, via stem cells usage [11–19]. Together with stem cell-based strategies, NGS can be improved to optimize patient selection, inform gene editing strategies, and increase the safety and effectiveness of individualized treatments [20, 21]. The basis of this review is the recent literature published in 2018–2024 and focused on the clinical use of stem cells in cancer precision medicine, which defines the multifaceted functions of stem cells in precision medicine and focuses on their application in cancer modeling, regenerative therapy, targeted drug delivery, and immunotherapy. It introduces the latest developments in therapeutic use, justifies the great difficulties in translation and ethics, and gives the future directions of the development of clinical implementation.

## Background

One of the pillars of the present biomedical science is stem cell research since it is likely to provide solutions to some of the diseases that cannot respond to conventional treatment. Stem cells are commonly identified by their undifferentiated cells that can self-renew and develop into specialized cell types [22–24]. The four most commonly identified major classes into which the stem cells are often differentiated based on their origin as well as potential are iPSCs, mesenchymal stem cells (MSCs), embryonic stem cells (ESCs), and hematopoietic stem cells (HSCs). ESCs are a pluripotent inner cell mass-derived blastocysts with a high differentiation capacity that serves as an ethically superior substitute to ESCs [16, 25–28]. MSCs, which are normally obtained by the isolation of umbilical cord blood, adipose tissue, or bone marrow, are multipotent and are commonly studied as tissue regenerators and immunomodulators [29–31]. HSCs, which are located in the bone marrow in large numbers, are the life-long blood and immune cells maker and the source of stem cell transplantation [32]. Table 1 shows a summary of the details of different stem cell types used in the treatment of cancer.

**Table 1:** Types of stem cells and their applications in cancer treatment

Stem cell type	Characteristics	Description	Applications for cancer treatment	References
Induced pluripotent stem cells (iPSCs)	Pluripotent, derived from somatic cells	Reprogrammed somatic cells to a pluripotent state	Personalized cancer models, drug testing, and genetic research	[16, 25, 27]
Mesenchymal stem cells (MSCs)	Tumor-homing, immune evasion	Multipotent cells can differentiate into various cell types	Targeted drug delivery, oncolytic virotherapy, and regenerative medicine	[33–36]
Hematopoietic stem cells (HSCs)	Hematologic regeneration	Stem cells from bone marrow that give rise to blood cells	Hematologic cancer treatment, immune cell engineering for therapies	[37–39]
Cancer stem cells (CSCs)	Tumor-initiating, therapy-resistant	A subpopulation of cancer cells with stem-like properties	Targeted therapies to eliminate CSCs and reduce tumor recurrence	[40, 41]

Although stem cells hold a lot of therapeutic potential, the constraints of traditional treatment options emphasize the importance of innovation. Conventional cures of chronic illnesses like cancer, neurodegenerative illnesses, and cardiovascular diseases frequently have limitations, which include low effectiveness, side effects, short-lasting action, and high recurrence rates. As an example, radiotherapy and chemotherapy may decrease the tumor size, but often they cannot eliminate cancer stem cells, resulting in recurrence [42]. Likewise, neurodegenerative disorders such as Alzheimer and Parkinson disease are incurable under the available pharmacological therapies, which at most have a transient symptomatic impact without reversing brain cell damage. Stents, bypass surgery, and pharmacological management of cardiovascular conditions improve the symptoms but fail to restore the injured myocardium [43–45]. Moreover, the last resort of organ failure, which is organ transplantation, is limited because of donors’ shortage, immune rejection and lifelong immunosuppression [46]. These limitations highlight the importance of research into regenerative approaches, and stem cell therapy appears as an appealing route for developing precision medicine.

## Translational applications of stem cells in cancer treatment

**Fig. 1:**
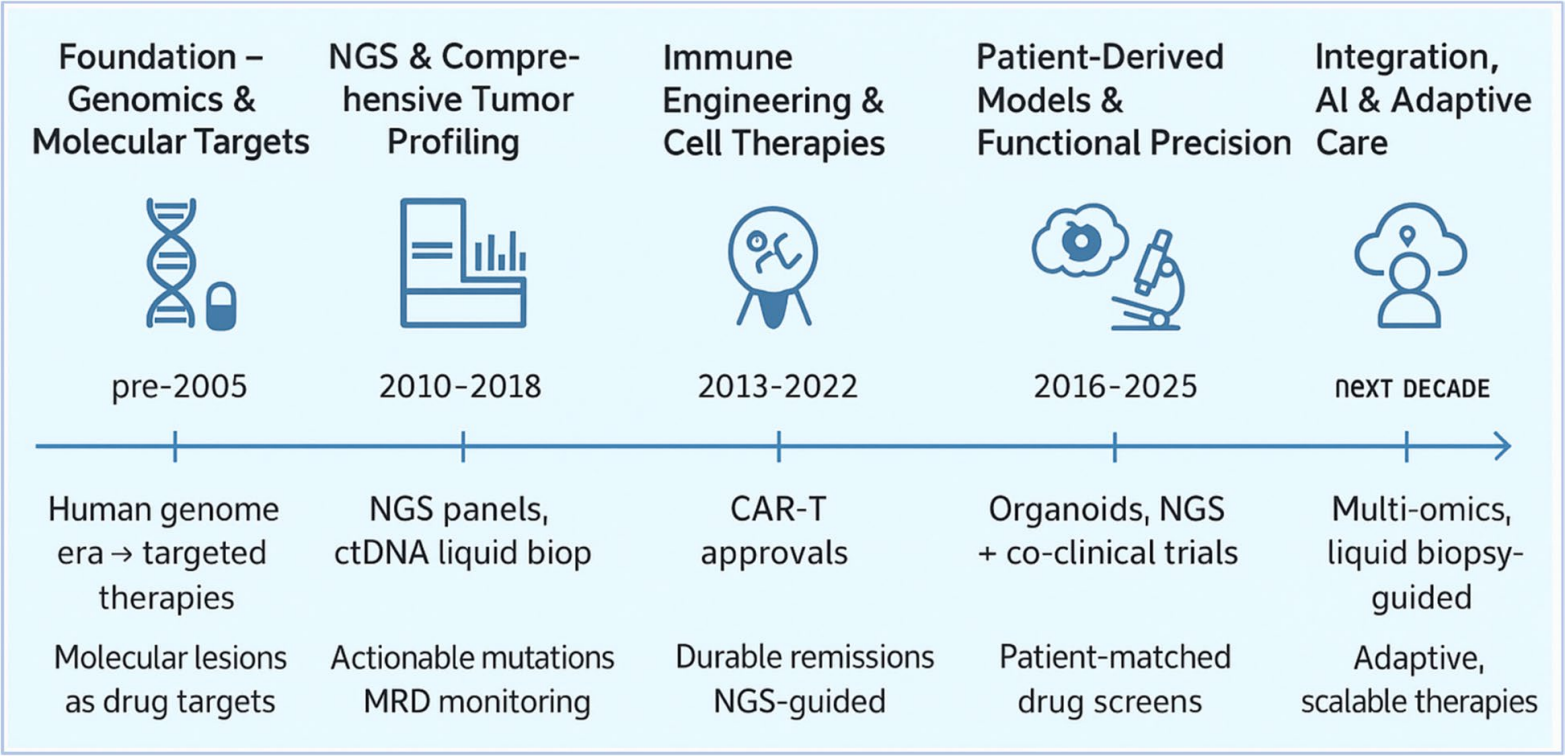
A timeline of precision oncology advancements, from genomic targets and NGS-based profiling to immune cell therapies, patient-derived models, and emerging integration with AI and adaptive care. MRD = minimal residual disease; ctDNA = circulating tumor; iPSC = induced pluripotent stem cells

In oncology and regenerative medicine, stem cells have been explored in different complementary modalities as treatment alternatives. Stem cell-based therapies offer long-term, specific, and patient-based cures as opposed to the traditional therapies that often exhibit systemic toxicity, low specificity, and high relapses. Figure 1 shows the broad insights into the advances in precision oncology over the decades.

### Targeting cancer stem cells (CSCs)

CSCs play a role in therapy resistance, metastasis, and disease recurrence. Stem cell-based approaches have been designed to selectively address CSCs by disrupting self-renewal pathways, by modulating the tumor microenvironment, or by amplifying immune-mediated clearance [47]. Indicatively, MSCs that are modified to release tumor necrosis factor-related apoptosis-inducing ligand (TRAIL) have been shown to be effective in preclinical models of glioblastoma and breast cancer and to eliminate tumor burden without destroying normal cells [48, 49]. HSCs have been demonstrated in other studies to play a role in immune reconstitution in the aftermath of high dose chemotherapy indirectly, which assists in CSC suppression [50, 51]. Also, in case of HSC transplantation, treatment methods including graft engineering have been able to curb chronic graft-versus-disease occurrence rates of 30–60% down to 7% and dramatically improve the quality of life in patients after transplantation [52]. These results underscore stem cells as a means of treatment as well as an adjunct that helps to increase the efficacy of the current modalities. It is essential to target CSCs during cancer treatment [47]. This can be addressed through targeting certain surface markers, such as CD44 and CD133, blocking of pathways, such as Wnt and Hedgehog, and immunotherapies, such as CAR-T cell therapy [40]. CSCs have proven to have potential in overcoming resistance and reducing relapse when targeted. However, there is limited clinical effect. Although initial results of ALDH1 inhibitor and CD44 blocker trials have positive interim results, toxicity and specificity are the significant challenges. This is indicative of the concerns in previous reviews that CSC markers are not completely tumor-specific, making therapeutic targeting tricky [53, 54]. A major challenge is thus the transition between preclinical efficacy to safe, long-lasting responses in patients.

### Patient-specific modeling with iPSCs

iPSCs are an innovative breakthrough in precision medicine as it enables the production of patient-specific pluripotent cells using somatic tissues. Their use in cancer therapy is based on their capacity to model tumor heterogeneity, screen drugs and potentially generate autologous cell therapies [37]. Evidence suggests that clinical research with iPSC derived immune cells may hold promise in hematologic malignancies as reprogrammed T cells and natural killer cells have demonstrated increased tumor-killing capacity [55, 56]. In particular, Takahashi and others have shown that the retinal cells derived by using iPSCs could be safely transplanted into patients which established a precedence of safety and feasibility in therapeutic execution [57–61]. Despite issues in guaranteeing genomic integrity and avoiding tumorigenicity, the preclinical and early clinical evidence indicates that iPSCs can help to bridge gap between individual modeling and therapeutic use. Table 2 lists the summary of significant studies that highlight advancements in the use of stem cells for cancer treatment, with a focus on personalized cancer models, oncolytic virotherapy, CAR-T cell therapy, and targeting cancer stem cells.

**Table 2:** A summary of key studies on stem cells in precision medicine and cancer treatment

Study area	Focus	Findings	References
iPSC models for cancer	iPSC-based personalized complex cancer models and iPSC-derived organoid replicates generation and applications	• Demonstrated the potential of iPSCs for creating personalized cancer models in terms of patient-specific tumors, responses to therapy • Drug delivery is improved using iPSC-derived organoid replicates of 3D tumors	[16, 25–28, 61–71]
Stem cell-based regenerative medicine	The potential of MSCs and HSCs in targeted delivery and regenerative medicine for targeting tumors and supporting repair in healthy tissues	• TRAIL-loaded MSCs effectively and selectively target metastasized cancer cells only • Bone marrow transplants derived from HSCs significantly improve treatment for hematologic cancer	[34, 35, 48, 72–81]
Precision vehicles for targeted drug delivery	Precision vehicle potential of MSCs for drug delivery to reduce off-target effects	• Chemotherapeutic agents and immune checkpoint inhibitors can be delivered with better accuracy using MSCs • MSCs effectively carry TRAIL to eliminate metastatic cancerous cells	[76, 82–85]
Targeting cancer stem cells	Targeting surface markers (e.g., CD44 and CD133), signaling inhibitors (e.g., Wnt, Hedgehog), and using CAR-T therapies to target CSCs	• CD44-targeted therapies reduce CSC populations and improve treatment outcomes • CSC-driven tumors are effectively removed using CAR-T therapy	[40, 41, 47, 86–89]
Combining immunotherapy and stem cells	• HSCs are used to generate immune cells for CAR-T cell therapy • Cytokines-engineered MSCs enhance immune improve responses to immunotherapy	• CAR-T cells engineered from HSCs show success in targeting specific tumor antigens • MSCs engineered to express immune-stimulating cytokines (e.g., IL-12) enhance the effectiveness of checkpoint inhibitors	[76, 90–98]

### Engineered stem cells as therapeutics

Genetic engineering has increased the therapeutic potential of stem cells, allowing them to serve as vectors of antitumor agents. One of the most clinically developed uses of engineered stem cells is to provide therapeutic payloads. Stem cells, especially MSCs, are promising to be targeted delivery vehicles because they are tumor-homing and immune-evasive [76, 82, 83, 99]. MSCs are developed to transport chemotherapy agents, immune checkpoint blockers, and oncolytic viruses to tumors [100]. As an example, MSCs expressing TRAIL were efficient to kill metastatic cancer cells, and MSCs modified to express immune checkpoint inhibitors improved anti-tumor immunity [76, 85]. The phase I trial of MSC-TRAIL therapy was feasible, with partial responses, although long-term follow-up information is not available to draw definitive conclusions on the survival benefit [82, 101]. In addition, early outcomes often capture response to treatment but often lack a measure of the experience patients have under care, hence, patient-reported outcome (PRO) inclusion will be essential in real-world benefit measurement [102–104]. Also, there can be manufacturing complexities, and regulatory hurdles that can restrict wider access. However, stem cell capacity to serve as simply intelligent carriers has a singular advantage over the traditional drug delivery systems. MSCs were also engineered to release prodrug-converting enzymes, cytokines, or oncolytic viruses that can directly operate in tumor niches. As an example, antitumor immune responses have been found to be better in solid tumor models with engineered MSCs expressing interleukin-12 [82]. Oncolytic viruses have also been used to selectively target gliomas using neural stem cells [105]. These methods highlight the flexibility of stem cells in providing site-specific delivery of drugs, reduction of systemic toxicity, and improving the therapeutic index of otherwise toxic agents.

### Integration of NGS with stem cell platforms

**Fig. 2:**
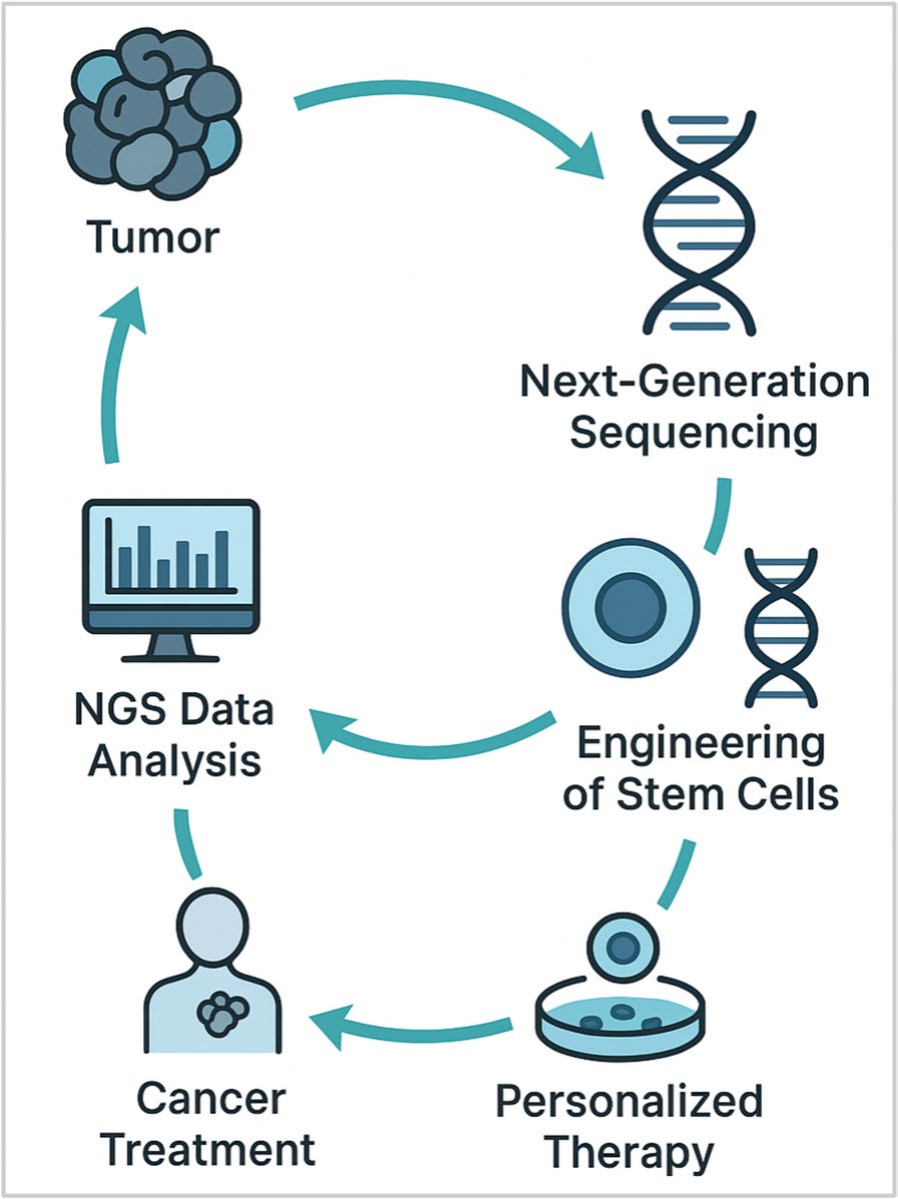
Workflow of precision oncology: tumor sequencing, NGS analysis, and stem cell engineering guide personalized therapy and treatment

NGS technologies have made it possible to further characterize the genetics of tumors and stem cell differentiation pathways. Figure 2 shows a general workflow of the integration of NGS in oncology. With the combination of NGS and stem cell systems, investigators could find patient-specific targets and predict a therapeutic response [21]. To take an example, intratumoral heterogeneity has been discovered through single-cell sequencing, which enables researchers to develop more efficient stem cell-based treatment [106, 107]. Furthermore, NGS enables the detection of minimal residual disease and mixed chimeras, allowing tailored monitoring and individualized patient care post-transplant. A study by Kim et al. (2025a, b) reported that low level mixed chimerism 2–5% and MRD were identified in 310 HSCT patients using CASAL NGS Assay. This were some of the strongest predictors of relapse (HR = 5.87), which supported the significance of NGS in targeted intervention [108]. A compilation of case studies published by AAICT from 2023 to 2025 showed that NGS-supported approaches demonstrated improvements in mobility in multiple sclerosis, communication in autism, and nerve regeneration in peripheral neuropathy/CIDP [109]. In addition, genomic profiling-based IPS models could be employed to filter drugs on the basis of mutations specific to a specific patient, which gives a new level of precision to therapy design. Besides that, studies have also revealed that lung cancer organoids integrating NGS data enabled the detection of druggable mutations within 2 weeks of the biopsy [110, 111]. This addition makes sure that the use of stem cells in oncology will not contradict the precepts of precise medicine.

### Emerging trends and translational potential

Some other possible applications of stem cells other than curing cancer are the use of tissue engineering, immune regulation and regenerative medicine. Checkpoint inhibitors in combination with stem cell therapy have the potential to overcome resistance to combination therapies. Further, stem cell 3D bioprinting demonstrates preliminary achievements in the production of complex tissue, which preconditions post-surgical cancer treatments [112, 113]. Synthetic biology is extending the list of engineered stem-cells that can perform complex therapeutic functions [114–116]. Collectively, these trends provide evidence of the transformational character of stem cell research, and its increasing combination with precision oncology and regenerative medicine. Table 3 covers a summary of recent research trends in stem cell cancer, such as combination therapies, use of organoids to test and personalize treatment, engineering stem cells to deliver drugs, and the development of CAR-T cell therapies.

**Table 3:** Summary of emerging trends in stem cell cancer research

Trend	Description	Implications for cancer therapy	References
Combination therapies	Combining stem cell-based approaches with existing therapies such as immunotherapy, chemotherapy, or oncolytic viruses	• Potential for synergistic effects, improving overall treatment efficacy • Potential reduction in drug resistance and relapse of tumor	[117]
Organoid models	Use of iPSCs- or CSC-derived organoids to model tumors in vitro and test therapies (personalized medicine and drug testing)	• Enhanced ability to predict patient responses and personalize treatment	[118]
Engineering MSCs for delivery	Modifying MSCs to deliver therapeutic agents or genes	• Targeted delivery systems can increase the specificity and effectiveness of therapies	[119]
CAR-T cell therapy	Engineering T cells to target specific cancer antigens	• Offers targeted destruction of cancer cells with minimal off-target effects	[120]
CSC-targeted therapy	Use of monoclonal antibodies, small molecules, or immune-based approaches to target and remove cancer stem cells	• Prevention of metastasis and recurrence of tumors • Improved long-term survival in cancer	[47]
Stem cell-derived exosomes	Stem cell-derived exosomes for tumor microenvironment remodeling, immunomodulation, and drug delivery	• A non-invasive option for delivering drugs • Improves immune response against cancer cells	[88]
CRISPR-based gene editing in stem cells	Use of CRISPR technology for modifying stem cells to enhance their therapeutic potential against cancer	• Improves precision of targeted therapies • Reduces resistance of cancer cells against treatment	[121]
Oncolytic virotherapy with stem cells	Direct delivery of oncolytic viruses to tumors using engineered stem cells like MSCs	• Efficacy of virotherapy enhanced in solid tumors • Immune clearance of oncolytic viruses reduced	[122]
Personalized stem cell therapy	Patient-specific stem cell-based treatment plans such as iPSC-derived immune cells or autologous CAR-T cells	• Reduced side effects and improved efficacy • Reduced risks of graft-versus-host disease	[97]
Bioprinting and 3D tumor models	3D bioprinting of complex tumor microenvironment for testing different therapies and conducting research	• More accurate depiction of the interaction between tumor and stroma • Advancements in precision medicine approaches	[123]

## Critical analysis

The reviewed evidence shows that stem cell technologies are becoming more applicable to the domain of precision oncology but their transfer to the routine practice is still in its infancy.

### Challenges in conventional treatment strategies

Despite a number of clinical breakthroughs and decades of research, cancer is still among the most conspicuous factors behind mortality in the world. Despite the successful survival of most types of cancer due to the use of traditional forms of treatment such as chemotherapy, radiation therapy, and immunotherapy, their failure has demonstrated a strong adverse effect not only in the rate of success but also patient satisfaction [124, 125]. The widely used conventional chemotherapy and radiation therapy are unspecific and have high risk of unwanted adverse outcomes on healthy tissues [42, 126]. Risks associated with chemotherapy can involve intra-tumoral heterogeneity and selection of a resistant subpopulation, particularly a slow-cycling, cell-cycle-sensitive cell that is hard to kill by the therapy [127, 128]. Additionally, medicines are frequently ineffective due to the lack of organization of vascular plexuses in tumors and the presence of the blood–brain barrier, which hinders the successful penetration of cytotoxic molecules to further reduce efficacy in solid tumors or brain metastases [129–131]. The acute and long-term toxicities including, but not limited to, nephrotoxicity, ototoxicity, neurotoxicity, myelosuppression, cognitive impairment and high treatment non-adherence lead to poor outcomes particularly in the elderly or underlying diseases populations of patients [132–134]. A review in 2025 found that about 40% of chemotherapy or radiotherapy patients faced cognitive decline, myelosuppression, and neurotoxicity. These negative outcomes add to the reported non-adherence rates of 20–30%, particularly among the elderly or comorbid patients [42]. In addition, patient-reported outcomes like quality of life can demonstrate that although conventional therapies are associated with clinical endpoints, they may still be insufficient in terms of maintaining physical and mental health, which is a gap that may be filled by stem cell-based therapies [103, 135, 136]. For instance, a systematic review published in 2022 reported that 50% of the patients who achieved clinical endpoints in their cancer treatments, such as tumor shrinkage, faced persistent adverse effects such as social isolation, emotional distress, and fatigue [137]. Table 4 outlines the main challenges, such as safety, efficacy, cost, and ethical concerns, associated with stem cell-based cancer therapies and the emerging opportunities that could enhance their clinical applications.

**Table 4:** Challenges and opportunities in stem cell-based cancer therapies

Aspect	Challenges	Opportunities
Safety	Risks of tumor formation and immune rejection	Advances in engineering and selection of safer stem cell lines
Efficacy	Variability in response and treatment resistance	Personalized approaches to tailor therapies to individual patient profiles
Cost	High cost of stem cell-based therapies and production	Potential for reduced costs with improved technology and scalability
Ethical considerations	Ethical issues related to stem cell sourcing and manipulation	Regulatory frameworks and ethical guidelines to ensure responsible research and use

### Clinical trials supporting stem cells in precision medicine

Initial clinical trials show promise of the translational potential of stem-cell-based strategies. As an example, the observation of long-term remission in leukemia and lymphoma patients with relapses following stem-cell-derived T cells engineered with chimeric antigen receptors has been reported [138, 139]. Mesenchymal stem cells reconfigured to express oncolytic viruses or gene-editing modified have demonstrated encouraging safety and targeting in glioblastoma and pancreatic cancer clinical trials [82, 90]. Likewise, induced pluripotent stem cell-derived natural killer cells are undergoing testing on their capacity to enhance tumor recognition without many off-target effects [140, 141]. The fusion of stem cells and immunotherapies, CAR-T cells, and checkpoint inhibitors, has enhanced cancer therapy further. HSCs are important in producing immune cells in CAR-T therapies against antigens such as CD19 in blood cancers [91–93, 95, 97]. MSCs that express cytokines such as IL-12 could increase the efficacy of immune checkpoint inhibitors [76, 90, 94, 96, 98]. Even with such developments, the majority of investigations are in preliminary stages, and substantial issues concerning scalability, immune compatibility, manufacturing cost, and long-term safety will need to be overcome before it can be incorporated into standard care (Table 5).

**Table 5:** Current challenges and future directions in stem-cell–based precision oncology

Category	Key challenges	Future directions
Clinical evidence	Most studies are in early phase with small cohorts and short follow-up; limited data on long-term safety and durable benefit	Large, multi-ethnic trials with extended monitoring to confirm efficacy and detect late toxicities
Manufacturing and access	Difficulties in producing stable, reproducible, and clinically viable stem cells; high costs and specialized infrastructure limit availability	Scalable biomanufacturing and cost-reduction strategies to broaden access beyond high-income regions
Tumor biology	Intra-tumoral diversity enables resistance to even personalized therapies; evolving cancer subclones reduce universal applicability	Incorporation of gene-editing and adaptive design approaches to better address heterogeneity
Immune environment	Suppressive tumor microenvironments diminish therapeutic response; immune evasion requires additional interventions	Combination approaches with immunotherapies or checkpoint inhibitors to enhance effectiveness
Safety risks	Potential for unintended tissue damage and off-target activity, particularly in immune-based stem cell therapies	Refinement of gene-editing tools (e.g., CRISPR) and synthetic biology platforms to improve precision
Ethical and regulatory issues	Debates around embryonic sources, patient-derived materials, ownership rights, and genetic privacy	Development of transparent ethical frameworks that ensure consent, equity, and responsible innovation
Data integration	Lack of real-world data reduces generalizability and patient-centered insight	Inclusion of patient registries and real-world evidence in trial design to inform broader application

### Ethical and regulatory frameworks

Stem cell-based interventions raise serious ethical and social issues. The international bodies, including the International Society of Stem Cell Research (ISSCR) point to the fact that pluripotent stem cells should be thoroughly controlled regarding their use, particularly when genetic editing is involved [142]. Genetic privacy is one of the core problems. There is a chance of genetic re-identification and misuse of genomic information with iPSC lines obtained from the existing patients and the patients themselves [67, 143]. It is necessary to use safeguards such as de-identification, informed consent databases and models, to provide ethical integrity. Moreover, the germline alteration is a problematic area that requires a rigid distinction between the novelty of the treatment and their application in reproduction [144]. Embryonic stem cell research remains an ethical concern with its destruction of embryos but iPSCs still remain a less problematic subject but have remain unresolved concerns on issues of consent of donors, ownership and long-term safety [145]. In addition to personal consent, the practical conditions of equitable access to high-technology therapies are acute, especially within a less-resource-intensive health care setting where cost and infrastructure can impose limits on the applicability of precision medicine using stem cells [146, 147]. These issues support the idea that the inclusion of ethics control and equity in the research structure and its clinical application translation are needed.

### Scientific and translational limitations

Despite the impressive potential of stem cell-based therapies, there are major obstacles. Cancer stem cell-targeting treatments display selective efficiency in preclinical models and fail to completely eliminate tumors, which has cast doubts on recurrence and resistance [148, 149]. On the same note, iPSC-based therapies, despite having patient-specific benefits, suffer the problem of genomic instability, tumorigenicity, and scalability to widespread clinical applications [150, 151]. Drug delivery—or immune modulation—engineered stem cells are technologically advanced but need stringent long term safety testing [152, 153]. With NGS becoming a part of stem cell research, there has never been a greater opportunity to gain a deeper understanding of the molecular world, but it is coupled with huge amounts of data that are challenging to interpret, to standardize, and to protect privacy [154]. These scientific and translational hurdles underscore the gap between the success of experiments and the ongoing clinical results.

## Ethical considerations and clinical translation

The implantation of stem cell technologies into the realm of accurate oncology poses deep ethical, legal, and social issues. Compared to the traditional approach to cancer therapy, the manipulation of patient-derived cells, the incorporation of genomic data and, in certain instances, technologies of germline editing are often required in stem-cell-based precision medicine [155, 156]. These require a robust set of ethics that ensure that the rights of patients, their privacy and security are upheld. Although the ethical issues associated with embryonic stem cells still occur, the application of iPSCs or engineered stem cells and mass genomic data have been given further consideration in the context of responsibility. In this regard, the issue of informed consent, the long-term follow-up of the patients, and ownership of the biological materials must be controlled [157]. There are systemic barriers to clinical translation, outside ethics. Regulatory approval procedures are not universal across regions and safety; efficacy and quality control can be regulated in diverse ways [158]. Many of the low and middle-income countries like Pakistan lack the infrastructure and resources to carry out highly sophisticated stem cell treatments [159]. These disparities raise the concerns of health equity in the world, where a tiny minority of patients can enjoy possibly life-saving innovations. Moreover, the incorporation of patient perspectives in the form of patient-reported outcome measures (e.g., FACT-G, EORTC QLQ-C30) is necessary to make sure that treatment assessment is based on the real-life patient health and well-being [160, 161].

## Limitations

Despite the accelerated developments in the field of stem-cell-based technologies, there are still many obstacles on the way to practice and the possibility to apply the stem-cell-based approaches to precision oncology. Most of the clinical trials that have been conducted so far are still at their early phases and tend to involve small number of patients with limited follow-up periods. This restricts the extrapolation that could be done on the long-term efficacy, endurance of action and even toxicity in the future [162, 163]. Additionally, the procedures of stem cells are hard to manufacture and standardize. Generation of stabilized, clinically grade stem cells that are stable, potent and genetically intact across laboratories is a major challenge [164]. These treatments are costly, and the facilities required to produce and distribute them pose a risk of only a small percentage of the patients being reached in a high-income setting, along with raising equity and globalizability concerns [165].

Biological barriers are also of great importance. Even generalized therapies based on stem cells are not possible because a range of subclones of cancer may become resistant to even the most personalized one [166]. There is also the problem of immune microenvironment which is likely to counteract the effectiveness of therapy and the need to combine the drugs with immunotherapies or with checkpoint blockers [167]. On-target effects in stem cell immune therapy are also more prone to causing unwanted tissue damage [168]. Ethical concerns that surround the use of embryonic stem cells, ownership of cell products, patient-derived products and ownership of cell products all introduce additional ethical dilemmas to clinical translation [169].

## Future directions

Large, multi-ethnic, long follow-up clinical trials are required to assure safety and generalizability. More affordable ways to reduce costs may include increasing accuracy and reducing off-target effects through the use of enhanced gene-editing systems such as CRISPR, and scalable biomanufacturing systems. With overcoming the prohibitive pricing of the stem-cell-based treatment, scalable bio-manufacturing living systems, automation of cell processing and generation allogeneic off-the-shelf stem cell lines may be less expensive and more readily available. The public–private partnership and global funding also may support the infrastructure that is subsidized and more accessible, i.e., not limited to high-income countries. Real patient data should be used in the design of the trial which will accelerate the learning process as well as focus on population-specific concerns. It is equally important to establish effective ethical and regulatory cultures that regulate consent, privacy and fair access in a manner that innovation does not outrun responsibility. It will require scientific, regulatory and societal collaboration in future to settle the problems of ethics and clinical translation. The future of this domain is in development of internationally recognized ethical and regulatory standards, development of safe and patient-controlled genomics data ecosystems, and development of large-scale, multi-ethnic, studies where generalizability of findings can be achieved. Precision oncology based on stem cells can become one step closer to becoming a groundbreaking and inclusive reality in cancer treatment through scientific rigor, ethical foresight and international collaboration.

## Conclusion

In conclusion, the idea of stem cell-based solutions is rather encouraging to create tumor-specific oncology, i.e., to address the issue of heterogeneity in tumors and enable more precise treatment. The results are still early and may be viewed with caution, but viability and therapeutic efficacy are possible according to preliminary clinical trials. Major challenges like biological complexity, barriers to production, excessive costs, and unaddressed ethical issues still exist. These limitations indicate that stem cell approaches despite their novelty are not ready yet to be applied in clinical practice. It will need further development with proper clinical trials, and advances in technology, and establishment of effective ethical and regulatory programs. Rather than a definite answer, oncology precision provided by stem cell research must be viewed as a dynamic science that, with time and collaboration, will be able to complement existing therapies of cancer and gradually change the pivotal approach to treating cancer.

## Data Availability

No datasets were generated or analyzed during the current study.

## Abbreviations

CSCs: Cancer Stem Cells
EGFR: Epidermal Growth Factor Receptor
ESCs: Embryonic Stem Cells
HSCs: Hematopoietic Stem Cells
iPSCs: Induced Pluripotent Stem Cells
MSCs: Mesenchymal Stem Cells
NGS: Next Generation Sequencing
NIH: National Institutes of Health
PPM: Personalized Precision Medicine
